# Modeling cell adhesion and proliferation: a cellular-automata based approach

**DOI:** 10.1186/s40323-015-0053-5

**Published:** 2015-12-02

**Authors:** J. Vivas, D. Garzón-Alvarado, M. Cerrolaza

**Affiliations:** National Institute of Bioengineering, Central University of Venezuela, Caracas, Venezuela; Department of Mechanical and Mechatronic Eng, National University of Colombia, Bogotá, Colombia; International Center for Numerical Methods in Engineering (CIMNE), Polytechnic University of Catalonia, Barcelona, Spain

**Keywords:** Cell adhesion, Computer simulation, Cellular automaton, Cell proliferation

## Abstract

**Background:**

Cell adhesion is a process that involves the interaction between the cell membrane and another surface, either a cell or a substrate. Unlike experimental tests, computer models can simulate processes and study the result of experiments in a shorter time and lower costs. One of the tools used to simulate biological processes is the cellular automata, which is a dynamic system that is discrete both in space and time.

**Method:**

This work describes a computer model based on cellular automata for the adhesion process and cell proliferation to predict the behavior of a cell population in suspension and adhered to a substrate. The values of the simulated system were obtained through experimental tests on fibroblast monolayer cultures.

**Results:**

The results allow us to estimate the cells settling time in culture as well as the adhesion and proliferation time. The change in the cells morphology as the adhesion over the contact surface progress was also observed. The formation of the initial link between cell and the substrate of the adhesion was observed after 100 min where the cell on the substrate retains its spherical morphology during the simulation. The cellular automata model developed is, however, a simplified representation of the steps in the adhesion process and the subsequent proliferation.

**Conclusion:**

A combined framework of experimental and computational simulation based on cellular automata was proposed to represent the fibroblast adhesion on substrates and changes in a macro-scale observed in the cell during the adhesion process. The approach showed to be simple and efficient.

## Background

The process of cell-substrate adhesion occurs on anchorage-dependent cells such as fibroblasts. This is accomplished either by electrostatic forces and other interactions of cell adhesion molecules on the cell membrane [1, 2]. The process involves initial events as protein adsorption, followed by cell adhesion and proliferation. Furthermore late events associated with cell growth, differentiation, and matrix deposition cell functioning are involved.

At the junctions between cell-substrate basal adhesions can be found. These adhesions are developed by anchoring proteins, mainly integrins, which allow the cell to adhere to the substrate through focal adhesions, connecting the actin cytoskeleton to the substrate through them [3, 4]. The bonding between cells and/or on a specific substrate determines the formation of a tissue. The functional properties of the tissue are also critically determined by the right ordering of the cells together. Hence understanding how the adhesion process and the factors involved in it is very important. A bad cell anchorage may trigger different pathologies which may appear in humans as disorders or physiological troubles.

The in-silico models can demonstrate how the biophysical stimuli can be correlated with the experimental patterns observed in the development of different tissues. One of these computational tools currently used is the cellular automata (CA). A cell population can be defined as a discrete dynamical system in space and time, where the individual behavior of each cell behaves according to specific variables and local rules [5]. Each element evolves according to its state at a specific time and the state of neighboring entities [6]. These states vary over time by changing stimuli from the cell or its neighbors [7].

Thus, this set of elements achieves a sensible evolution towards the state of neighboring elements which is known as the local transition rule. This rule affects a number of states within the regular arrangement, known as the evolution of space and determines the behavior of cellular automata [8–10]. Within this context, several authors have developed algorithms in which the cells are considered as elements of an automaton whose evolution rules were established by the behavior of normal cells and cancer cells [11–13].

Another cellular-automaton model is developed in [14], in which the cell behavior is regulated by oxygen concentration, thus involving the use of nutrient-transport equations in the model. The main objective was to study both cell migration and growth within scaffolds in-vitro. Moreover, for the study of cell proliferation process simulations were carried out in a two-dimensional network of the finite state defined by the automaton [15]. In these works the authors were based on a single cellular process at a time.

Therefore, to understand the cell dynamics during its life cycle, a cellular-automata model for the cell adhesion process of fibroblasts on a substrate, followed by cell proliferation, was designed and implemented in the present research. For this model, the work is carried out in two steps: the first one focused on experimental trials of the 3T3-fibroblast cell-culture to assess the adhesion process and the second part consisting in the computational simulation using cellular automata.

## Methods

As mentioned above, the work is carried out in two steps in parallel. The experimental part show the changes that occur in the cell after the binding process to the substrate begins, followed by the cell proliferation, while the second part carries out a computer simulation using cellular automata.

### Experimental tests

#### Establishment of the 3T3cell culture

A 3T3 cell culture of murine fibroblasts was used. These cells were cultured from cryo-preserved cells in liquid nitrogen. Cells were thawed at 37 $^{\circ }$C in thermostat bath, centrifuged and resuspend in culture medium DMEM-F12 supplemented and kept in an oven at a temperature of 37 $^{\circ }$C and an atmosphere of 5 % CO$_{2}$ and 90 % humidity. After reaching a confluency close to 80 % in a T-75 flask cell count was performed using Trypan-blue and then resuspend in a medium DMEM-F12 supplemented with 10 % fetal bovine serum and 1 % antibiotic-antimycotic. The rest of the trials were carried out with this cell population.

#### Cell adhesion evaluation

The evaluation of the adhesion process was performed through continuous observation with an optical microscope. Approximately 1 $\times$ 10$^{4}$ cells were plated in a T-25 flask, ensuring that they were dispersed enough to permit the focus on few individual cells without forming cell aggregates. The cell declination was recorded by taking pictures every 30 s for 5 min. Later the cells on the flask surface were photographed every 5 min for a period of 3 h. Tests were performed in triplicate.

#### Cell proliferation

Once the cells are sedimented on the bottle surface the cell viability and proliferation during 24 h-periods was determined. The culture medium was removed and 100 $\upmu$l of MTT (concentration 5 mg/ml) and 400 $\upmu$l of culture medium were added. Then, it was incubated for another 3 h. Later the supernatant was removed and 1 ml of DMSO (dimethyl sulfoxide) was added to dissolve the formazan generated at intracellular level [16]. It was allowed to solubilize for 5 min and was read with a PERKIN-ELMER brand spectrophotometer at a wavelength of 570 nm. The test was performed in quadruplicate.

### Computational model

**Fig. 1:**
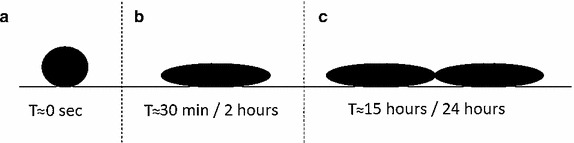
Schematic representation of the cell adhesion and proliferation processes

In order to simulate the adhesion process in time for a generic cell population a computational model based on cellular automata is proposed herein. While the first step of the process occurs in seconds (see Fig. 1a) the cell adhesion was completed after 2 h (see Fig. 1b). Finally new cells can be observed after 24 h, as shown in Fig. 1c.

A cellular-automata methodology considering each cell as an individual entity is employed. The simulated area is a generic space whose lower edge is smooth, with a length of 1 mm and a height of 0.5 mm.

A rectangular domain was defined and discretized with square elements. Thus, a regular mesh of 15 $\upmu$m-sided quadrilateral-elements was defined, having 67 horizontal and 33 vertical elements. The cell is considered as a two-dimensional 15 $\upmu$m-diameter spherical structure in suspension with defined and constant mass and volume. The simulation started with 50 initial cells randomly placed. This number of cells guarantees an available area in the bottom surface of the simulation domain, which represents the substrate where the cell will lie and anchorage.

**Fig. 2:**
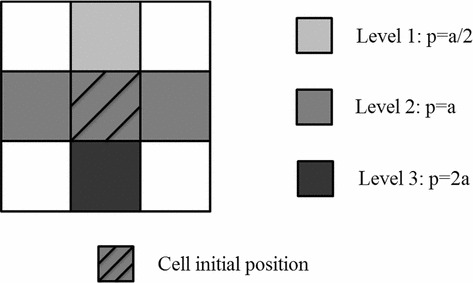
Representation of the cell neighborhood and probabilities of its new position according to level 1, 2 or 3

To implement the CA model is necessary to define a neighborhood around the element. This delimits the adjoining set of cells and relative position to each of them as a spatial arrangement of pixels, where each participant pixel has an influence on one or more pixels within the spatial arrangement. Starting from an initial state of the cell population, they change their states synchronously at each instant. This change is established via a neighborhood system of Von Neumann, as can be observed in Fig. 2. A free and open boundary is considered in the model where all the cells are allowed to be outside this boundary. Then the number of cells in the model is known. The number of cells can decrease when a cell approaches the boundary limit and thus it becomes out of the cells population.

The probabilistic aspects of the simulation must be considered within its dynamics. As well, further studies are also being done to consider other neighborhoods such as the system Moore.

The state of a cell in a given generation depends upon the states of neighboring cells and its own state in the previous time. The time is discrete and during the progressive steps the space is partitioned into discrete cells and conditions can be defined in a finite space. At the beginning (t $=0$) the cell occupies a random position in the space, which is denoted as P$_{0}$ (position 0). Then, from this state, possible places that the cell can occupy in the next time step are defined. The next position of the cell is given by chance together with the evolution rules of the method. The cells are assumed to be in a good physiological shape in the G0 state of the cell cycle. This guarantees the continuity of the cell cycle once the cell made contact with the substrate surface.

Any design can be set as an initial condition in a given time t$_{0}$ and every cell of simultaneous order has a value involving a new global state at time t$_{1}$. Then, the new value of a given cell at time t is a function of the values and locations of the cell which are the neighborhoods found at time t$_{0}$. Therefore a sequence of global states is formed for its interaction each other which is usually known as the transition function.

The CA has the ability to detect collisions between cells (defined as the evolution rule). Thus the cell can move forward in time, reaching new positions in the neighborhood without preventing the displacements of closer cells (two cells can’t occupy the same physical space). In this model, we are describing just the falling down of the cells. We can represent the inlet and outlet of cells and to avoid the possibility that cells coming from the adjacent non-considered domain become part of the modeled domain we have included a reflexive boundary. On the other hand, cells cannot move in diagonal directions since a von Neumann neighborhood scheme was chosen for the CA model.

The factor that is directly involved in the cellular sedimentation is the gravity since the culture system is stationary and no disturbances are observed in the medium. The sedimentation velocity of the cell into the medium is estimated by the Stoke’s law which calculates the movement of small spherical particles moving at small velocities as

1 $$\begin{aligned} Fr=6\pi R\eta v \end{aligned}$$

where *R* is the radius of the sphere, *v* is the velocity and his the fluid viscosity. Then for particles falling down within a viscous medium because of their own weight, the velocity of sedimentation can be computed by equaling the drag force with the apparent weight of the particle in the fluid as shown below

2 $$\begin{aligned} v=\frac{\frac{2}{9}r_c ^2( {\rho _c -\rho _m }).g}{\eta } \end{aligned}$$

being r$_{\mathrm{c}}$ the cell radius, $\rho _{\mathrm{c}}$ the cell density, $\rho _{\mathrm{m}}$ the medium density, $\eta$ the medium viscosity and g is the gravity constant 9.8 m/s$^{2}$. This value determines the speed with which the cell descends over time. To compute the probability associated to every cell decline, the vicinity of each cell is divided into three levels, where each level has a different probability of being occupied in the next time step. Level 1 consists of a single possible position above the initial location of the cell. Level 2, with two possible positions for the new location of the cell, has an associated probability p $=$ a while the lower level, level 3, was assigned a probability p $=$ a/2 as shown in Fig. 2.

To assign this sedimentation condition to the simulation the “a” value is determined below. The sum of the probabilities of the 3 levels in the vicinity is given by

3 $$\begin{aligned} a/2+a+2a=1 \end{aligned}$$

and therefore

4 $$\begin{aligned} a=2/7 \end{aligned}$$

Now, by substituting the value of the probability (Eq. 4) in the neighborhood already established levels (see Fig. 2) the probabilities for a new position of each cell are: $\mathrm{p}_{(\mathrm{level}\,\,1)}=\mathrm{a}/2=1/7$ (the lowest probability) to be less likely that the cell ascends in time; p$_{(\mathrm{level}\,\, 2)}$ $=$ a $=$ 2/7 and finally p$_{(\mathrm{level}\,\,3)}$ $=$ 2a $=$ 4/7 which has the highest probability of being occupied. Here, the gravity is the determinant factor.

**Fig. 3:**
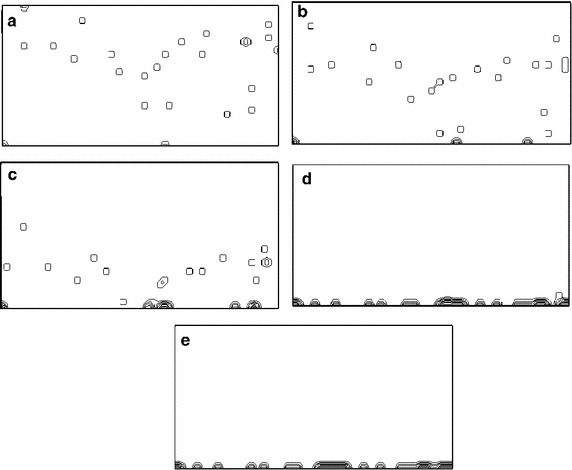
Cells sedimentation during computer simulation (*side views*). **a** Cells initial positions at t $=$ 0. **b** First cell in contact with the substrate at t $=$ 4 s. **c** Cells are in the lower half of the simulation area at t $=$ 10 s. **d** All the cells contact at least one surface at t $=$ 22 s. **e** All the cells are over the substrate at t $=$ 34 s

In cases where the new selected location is occupied by another cell, the system randomly chooses a new position. In cases where there is no free space for movement, the cell will take the same place in the next time step. This selection of the new location is performed as often as necessary until the cell finds a position on the contact surface, defined inside the borders of the simulation environment.

#### Cell adhesion model

Once the cell is above the surface the neighborhood changes to two possible side positions, again randomly selected. If any of the side positions is available the cell begins the adhesion process that is simulated as a morphological change, where the cell will occupy two continuous positions in the same time interval. If the new position is occupied by another cell, the searching for available positions to the cell is performed.

#### Cell proliferation model

When the cell is fully adhered the proliferation process starts. A new search of side positions around the cell is performed and a third neighborhood of the adhered cell is defined. If two continuous free side-positions are available then the cell duplicates. Otherwise the cell is not duplicated and a contact inhibition phenomenon is presumed.

## Results

For the simulation, 50 initial cells were placed in a 0.5 mm$^{2}$ simulation environment. The initial position of all cells in the computational model was randomly assigned.

### Celular sedimentation

The theoretical velocity of sedimentation obtained using Eq. (2) was *v* $=$ 8.5 $\times$ 10$^{-5}$ m/s. Furthermore simulation predicted a sedimentation time of 22 s for a cell population of 50 starting cells, randomly placed on the simulation environment (see Fig. 3a). During the fall of the cells, each iteration requires 0.5 s. As the position of each individual cell is randomly assigned, drop times are different for each cell, depending on the distance to the substrate they were at t $=$ 0 s. The first cell comes into contact with the substrate after 4.0 s (see Fig. 3b). After 44 iterations and 22 s, as shown in Fig. 3d, the cells have descended and made contact with at least one surface. It can be noted that there exist still cells that are not in contact with the bottom surface (see the cell very close to the bottom, at the right zone of the figure). Also, at the middle bottom region of Fig. 3d, it can be seen a cell with a like-spherical shape, before to attach to the surface. Finally, at 34 s the entire cell population is completely attached to the substrate, as displayed in Fig. 3e.

### Cell adhesion

**Fig. 4:**
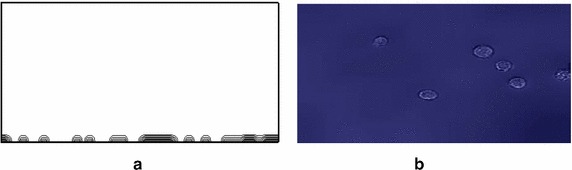
Final position of the cells over the substrate at t $=$ 34 s. **a** Side view of the simulation. **b** Micrography of in-vitro test. $\times$20 (*top-to-bottom* view)

In Fig. 4, the simulated cells on the contact surface in the initial phase of adhesion (focal adhesion) are observed. The adhesion process begins once the cell contacts the substrate. The adhesion times vary since cell descended at different times according to the distance to the substrate. Initially the cells are observed with their characteristic rounded shape with sharp edges, representing the cytoplasm concentrated in the spherical shape of the cell. The morphological change of the cells occurs in about the first hour after the first contact with the substrate. The model required 236 iterations to simulate the adhesion process.

**Fig. 5:**
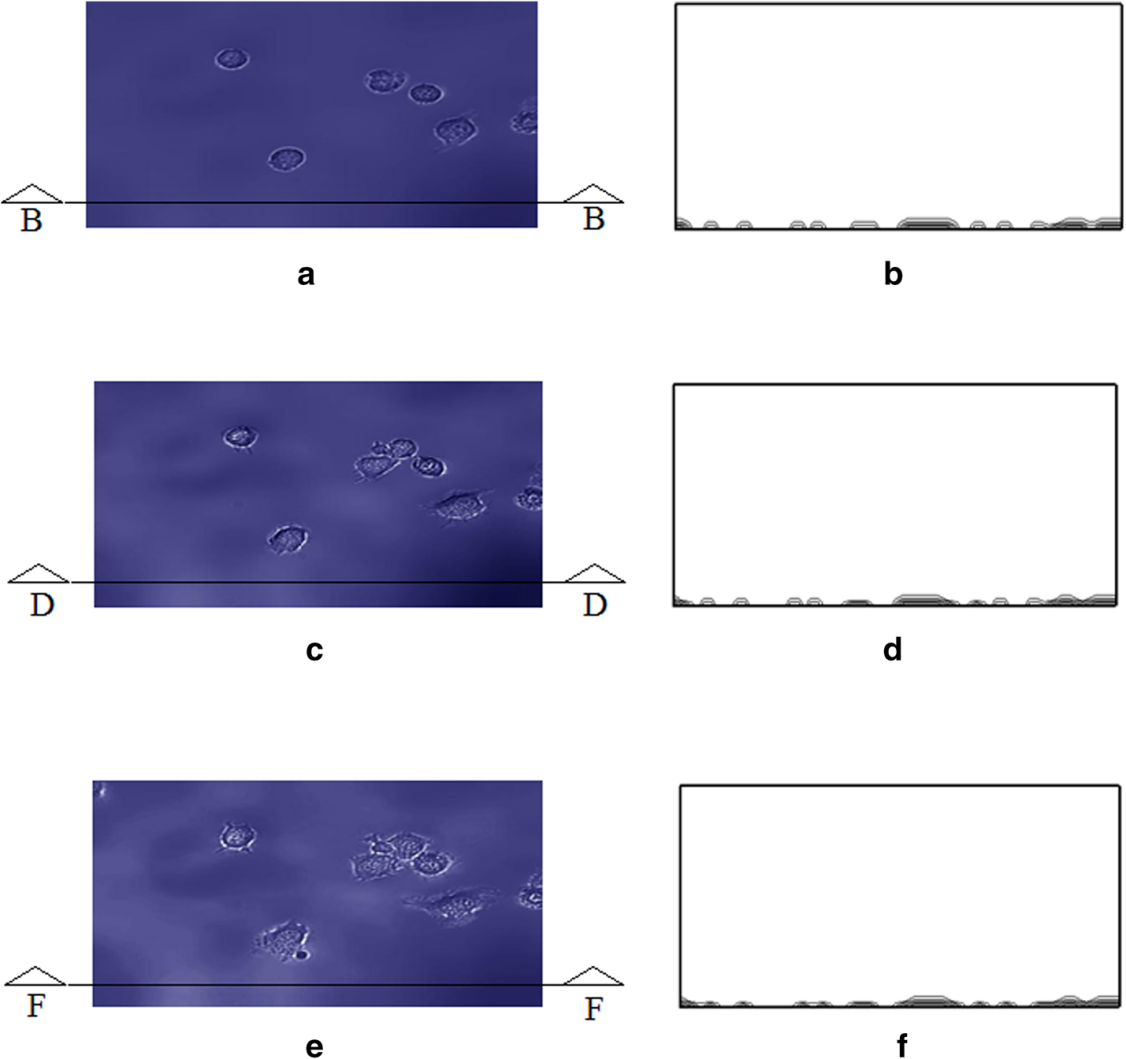
Cell shape variation during the adhesion process. Images on left column (**a,c,e**) come from in-vitro testing (top-to-bottom views) while images on right column (**b,d,f**) come from computer simulations (side views of the environment). *Upper row:* morphological changes of the first cells at t$\approx$100min. *Middle row:* shape changes in intermediate cells at t$\approx$145min. *Lower row:* shape changes in all cells at t$\approx$180min

In Fig. 5, the change in cell morphology can be seen as time progresses. After approximately 80 min (240 iterations of the computational model), the more cells are adhered to the substrate. Cells with cytoplasmic projections are observed, losing their initial spherical shape. Figures in the left column of Fig. 5a, c and e display a top-to-bottom view of the experimental results, while figures in the right column (b, d and f) represent a lateral view (in two dimensions) of the computational simulation corresponding to experiments reported in figures a, c and e.

**Fig. 6:**
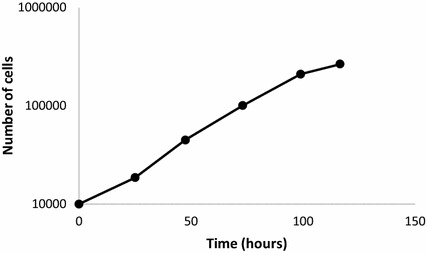
Evolution of the number of 3T3 cells in the culture

The formation of the first extensions of the cytoplasm in a cell are observed in Fig. 5a, b, suggesting that this entity was the first to contact the substrate and the first to begin the anchoring process. These elongations occur at 100 min after the cell decline. After 145 min (equivalent to 540 iterations) a scattered cytoplasm with a larger number of lateral projections is observed, also missing the initial spherical shape of the cells, as shown in Fig. 5c, d.

The adhesion process is then completed after about 180 min, showing spindle-like-shape cell morphology which is characteristic of this cell type. The cytoplasm occupies the maximum space in the substrate, allowing neighboring cells to interact with each other as displayed in Fig. 5e, f.

### Cell proliferation

The increase in the number of cells as time progresses is observed in Fig. 6. The culture was started with 1 $\times$ 10$^{4}$ cells and after 24 h the cell population doubled successfully. Cell number continues to increase in the next 100 h achieving a slight *plateau* which can be related to the area available for the culture. This behavior suggests the limited presence of resources to maintain the proliferation rate. Once the culture reaches a semi-confluent condition the area available for growth is limited, thereby decreasing the rate of proliferation. Upon reaching the confluence contact inhibition is observed in the cells after 120 h in culture, reflected as a steady-state in the cell proliferation.

Figure 6 shows a population of cells in a plate. This figure does not have a strict correspondence with the computational model because the growth plate represents a 3D experimental (in-vitro) model while the computational model is a 2D model whose domain is a cross-section of the 3D model. In addition, Fig. 6 displays the whole well plate instead of the 2D model that represents a window of dimensions 1.0 mm width $\times$ 0.5 mm height. This is a representative volume close to the well-plate surface. It should be mentioned here that, in order to refine this simulation, a multiscale model is currently being developed, which is oriented to the representation and prediction of the cell colonization in a spherical scaffold.

**Fig. 7:**
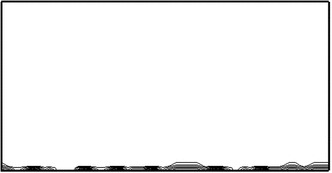
Duplicated cells covering the whole contact surface

In our model, all cells attached and having extended morphology on the substrate begins the cell proliferation process. This computational domain represents a rectangle cross-section close to the surface of the well plate where the events occur. Analogous to the experimental tests, the cells with side free positions in substrate duplicated (Fig. 7). The proliferation can be seen with the surface completely covered by cells, which are graphically observed when looking at all occupied positions of the lower part of the cellular-automata mesh. Both initial and “daughter” populations are observed as well.

Conversely, in those cases where two or more cells are adjacent each other at the surface, the proliferation process fails to start. When the whole substrate surface is covered by cells, the cells halt their proliferation process in a phenomenon known as contact inhibition.

## Discussion and concluding remarks

Cell adhesion and cell proliferation were modeled and described in this work by a using CA computational model. This 2D model represents the falling down and lying of the cells when they are attaching to a surface.

The sedimentation time in the simulation corresponds to the numerical value obtained theoretically by Eq. (3). The values of the variables involved were established according to the real conditions of the culture system. The behavior observed in the simulation is due to the conditions provided to the system, where each cell behaves individually also being able of independent movement but limited because of the positions of the other cells. These parameters are specific to the cell type tested and the culture conditions used.

While the cells tend to move down because of the gravity there is a low probability of a lateral movement which delays the cell declination. These variables were included in the simulation. Studies conducted in previous decades have shown that cells recognize each other and become adhered in specific ways [17, 18].

In our simulation, the adhesion process begins once the cell is on the substrate. This process is based on the presence of specific molecules called “Cell Adhesion Molecules” (CAMS) [19]. The formation of a link between a cell and the solid surface is dependent on the force exerted by both structures in contact [20].

The model showed the two steps of the adhesion process in a cell population. The formation of the initial link between cell and substrate, which occurs in the first minutes of the adhesion, known as focal adhesion, was observed after 100 min where the cell on the substrate retains its spherical morphology during the simulation. Immediately after, the reorganization of the molecules of the membrane occurs. Also the concentration of cytoskeletal in adhesive regions and the reorganization of glycocalyx occur to minimize repulsion between the cells and the substrate [21, 22]. This process allows the maturation of focal adhesions, then increasing the number of anchoring sites and making adhesions. The size and distribution of these adhesions reflect the contractile state of the cell [23].

The morphological change of cells after at least 1 h after the starting of the adhesion process is observed. This phenomenon is dependent on the space available, allowing the cytoskeleton to be strongly anchored to the substrate. Moreover, the adhesion process is not reversible and once started, the cell is capable of activating mechanisms and signaling cascades that allow the proliferation to start [24].

In a general sense, it can be observed the adhesion process caused by the change in the cytoplasm. During this process the cytoplasm extends, retaining its initial volume and occupying the maximum space, as shown in Fig. 5d, f. This cytoplasm extension is related to the presence of a set of integrin receptors responsible for the binding of substrate to the cytoskeleton [25]. At this step, the glycoproteins of the cell membrane are adsorbed to the surface. The cell deformation takes place while it tries to occupy the largest possible area. Particularly, the 3T3 cells in suspension without contact with any surface do not duplicate. This phenomenon is commonly known as a “anchorage-dependence” on the cell division.

The frequency at which the cell duplicates is increased as cell spreads. This fact could be associated with the fact that the more extended cells with larger surfaces are, the greater the number of growth-factors molecules and nutrients they can capture. The cells of the 3T3 cell line are, however, virtually unable to grow in suspension but they can duplicate rapidly when they find an anchorage point in any surface by forming a focal contact. Even if this anchorage point is so small for preventing the cell extension, it will enable them to duplicate more frequently.

This focal contact occurs in the first seconds of contact between the cell and substrate surface. As time progresses these initial adhesions mature, get stronger and in greater quantity that allows a better attachment of cells. These processes also allow the cell to be anchored on the surface, giving it the right signaling for the cell to advance from G1-step to the S-step of the cell cycle [26, 27]. The signal intracellularly emitted by integrins acts in close synergy with those signals produced by growth factors thus helping in the cell evolution.

The total colonization of the surface is determined by the ratio of available area and the number of initial cells being considered. In our test it was observed that the cell population doubled after 24 h, corresponding to results reported in the literature [28–30]. The cell duplication process is progressive which allows the new cell to remain anchored to the surface. Furthermore cytokinesis requires the action of integrins because if they are inactive separation of daughter cells will not occur. It is also presumed that integrins are anchor points to generate traction forces. Both cadherins and integrins (adhesion molecules) have been related to the orientation of the division arc that will separate the two cells. Hence the importance of evaluating both processes in a continuous manner since the cell proliferation in general requires cell anchorage. These results could be extrapolated to larger surfaces as well as to different cell types since the adhesion behavior in initial steps is similar for all cell types.

However, both adhesion and proliferation times as well as lifetimes, proliferation and even lifetimes must be considered since they play a determinant and specific role in the substrate conditions under study. Similarly, the study of cell proliferation can be potentially used to quantify or optimize experimental tests that require large number of cells thus reducing costs in estimating the cellular behavior.

There reader can find some other works which have proposed modeling of cell adhesion and proliferation. Among these applications, it should be mentioned the modeling of different kind of cells, e.g., for cancer [31, 32] and different extracellular matrices [33]. The adhesion and proliferation of cells in fibrin scaffolds, however, have not been previously modelled. Indeed, the model proposed herein is a simple starting point for this kind of models.

Finally, it should be mentioned herein that our computational model is an approximation to the experimental testing environment because cells are constrained to move in a plane system xy while in the real experiment they move in a space xyz. Therefore, our model can be viewed as a starting point for more realistic approaches. Also, it should be mentioned here that, in order to refine this simulation, a multiscale model is currently being developed, which is oriented to the representation and prediction of the cell colonization in a spherical scaffold.
